# Persistent epigenetic signals propel a senescence-associated secretory phenotype and trained innate immunity in CD34^+^ hematopoietic stem cells from diabetic patients

**DOI:** 10.1186/s12933-024-02195-1

**Published:** 2024-03-29

**Authors:** Maria Cristina Vinci, Sarah Costantino, Giulia Damiano, Erica Rurali, Raffaella Rinaldi, Vera Vigorelli, Annalisa Sforza, Ermes Carulli, Sergio Pirola, Giorgio Mastroiacovo, Angela Raucci, Assam El-Osta, Francesco Paneni, Giulio Pompilio

**Affiliations:** 1 Unit of Vascular Biology and Regenerative Medicine, Centro Cardiologico Monzino IRCCS, Via C. Parea 4, 20138 Milan, Italy; 2 Center for Translational and Experimental Cardiology (CTEC), Department of Cardiology, University Hospital Zurich and University of Zürich, Zurich, Switzerland; 3 Dipartimento Di Scienze Cliniche E Di Comunità, Università Di Milano, Milan, Italy; 4 Doctoral Programme in Translational Medicine, Università Di Milano, 20122 Milan, Italy; 5 Department of Cardiac Surgery, Centro Cardiologico Monzino IRCCS, Milan, Italy; 6 Unit of Experimental Cardio-Oncology and Cardiovascular Aging, Centro Cardiologico Monzino IRCCS, Milan, Italy; 7 Epigenetics in Human Health and Disease Program, Baker Heart and Diabetes Institute, Melbourne, VIC 3004 Australia; 8 Department of Biomedical, Surgical and Dental Sciences, Università Degli Studi di Milano, Milan, Italy; 9 University Heart Center, University Hospital Zurich, Zurich, Switzerland

**Keywords:** Diabetes mellitus, Cardiovascular disease, Trained immunity, Epigenetics, Hematopoietic stem cells

## Abstract

**Background:**

Diabetes-induced trained immunity contributes to the development of atherosclerosis and its complications. This study aimed to investigate in humans whether epigenetic signals involved in immune cell activation and inflammation are initiated in hematopoietic stem/progenitor cells (HSPCs) and transferred to differentiated progeny.

**Methods and results:**

High glucose (HG)-exposure of cord blood (CB)-derived HSPCs induced a senescent-associated secretory phenotype (SASP) characterized by cell proliferation lowering, ROS production, telomere shortening, up-regulation of p21 and p27genes, upregulation of NFkB-p65 transcription factor and increased secretion of the inflammatory cytokines TNFα and IL6. Chromatin immunoprecipitation assay (ChIP) of p65 promoter revealed that H3K4me1 histone mark accumulation and methyltransferase SetD7 recruitment, along with the reduction of repressive H3K9me3 histone modification, were involved in NFkB-p65 upregulation of HG-HSPCs, as confirmed by increased RNA polymerase II engagement at gene level. The differentiation of HG-HSPCs into myeloid cells generated highly responsive monocytes, mainly composed of intermediate subsets (CD^14hi^CD16^+^), that like the cells from which they derive, were characterized by SASP features and similar epigenetic patterns at the p65 promoter. The clinical relevance of our findings was confirmed in sternal BM-derived HSPCs of T2DM patients. In line with our in vitro model, T2DM HSPCs were characterized by SASP profile and SETD7 upregulation. Additionally, they generated, after myeloid differentiation, senescent monocytes mainly composed of proinflammatory intermediates (CD14^hi^CD16^+^) characterized by H3K4me1 accumulation at NFkB-p65 promoter.

**Conclusions:**

Hyperglycemia induces marked chromatin modifications in HSPCs, which, once transmitted to the cell progeny, contributes to persistent and pathogenic changes in immune cell function and composition.

**Supplementary Information:**

The online version contains supplementary material available at 10.1186/s12933-024-02195-1.

## Introduction

Diabetes mellitus (DM) is a major global health problem with 537 million affected individuals in 2021 that is predicted to rise to 783 million by 2045. Diabetes-related cardiovascular complications represent one of the world’s leading causes of morbidity and mortality, with $327 billion of total annual cost, only in the US (International Diabetes Federation. IDF Diabetes Atlas, 10th edn. Brussels, Belgium: 2021. https://www.diabetesatlas.org). The common “leitmotif” underlying cardiovascular disease initiation and progression in DM patients is chronic inflammation, a condition in which immune cells, namely monocytes/macrophages play a major role [1]. As components of the immune system, monocytes arise from hematopoietic stem/progenitor cells (HSPCs) in a process called hematopoiesis, taking place in a specialized bone marrow microenvironment known as the HSPC niche [2]. DM was shown to alter hematopoiesis considerably [3] by promoting myelopoiesis and the generation of monocyte subsets characterized by an inflammatory, pro-atherogenic phenotype eventually leading to vascular dysfunction and atherothrombotic complications [4–6]. A series of experimental studies have recently demonstrated that the diabetic environment fosters pro-inflammatory transcriptional programs leading to immune cells with pathological phenotype by chromatin modification [7, 8]. In this regard, epigenetic modifications have been reported to promote the upregulation of genes implicated in inflammation and monocyte-derived macrophage polarization in mouse models of diabetes [9–11]. Furthermore, while H3K4me1 regulation is implicated in mouse embryonic stem cell differentiation, the role of SETD7 in HSPCs remains poorly understood [12]. Although these studies provided important insights into the role of epigenetic changes in this setting, it remains unknown whether epigenetic signals that participate in immune cell activation and inflammation already take place at the level of bone marrow and whether they can be transferred to more differentiated immune cells during replication in humans. We and others have previously shown that hyperglycemia induces long-lasting changes of the epigenetic landscape which persist even after normalization of glucose levels, both in vitro and in vivo [13, 14]. Specifically, in vascular endothelial cells glucose spikes were found to induce specific chromatin modifications (i.e. SETD7/H3K4me1 activation) and subsequent alterations of transcriptional programs implicated in endothelial inflammation [14, 15]. This phenomenon, defined as “hyperglycemic” or “metabolic memory”, might contribute to explain the persistent burden of cardiovascular disease despite optimal glycemic control, as shown in recent clinical trials [16].

Here, we hypothesize that epigenetic changes occur early in HSPCs and that—through the “metabolic memory” phenomenon [13, 14, 17]—they are transferred to the myeloid progeny thus fostering a proinflammatory phenotype in mature immune cells. Hence, the present study was designed to address the following goals: (1) to assess whether in vitro exposure to hyperglycemia, a hallmark of DM, alters myeloid cell differentiation via epigenetic “priming” of HSPCs; (2) to determine whether such epigenetic modifications are transferred to the differentiated cell progeny; (3) to assess if similar mechanisms are at work in HSPCs isolated from the bone marrow (BM) of patients with type 2 diabetes (T2DM).

## Methods

A full description of the methods used for this study can be found in the Additional file 2 and 3.

### Study design

**Fig. 1:**
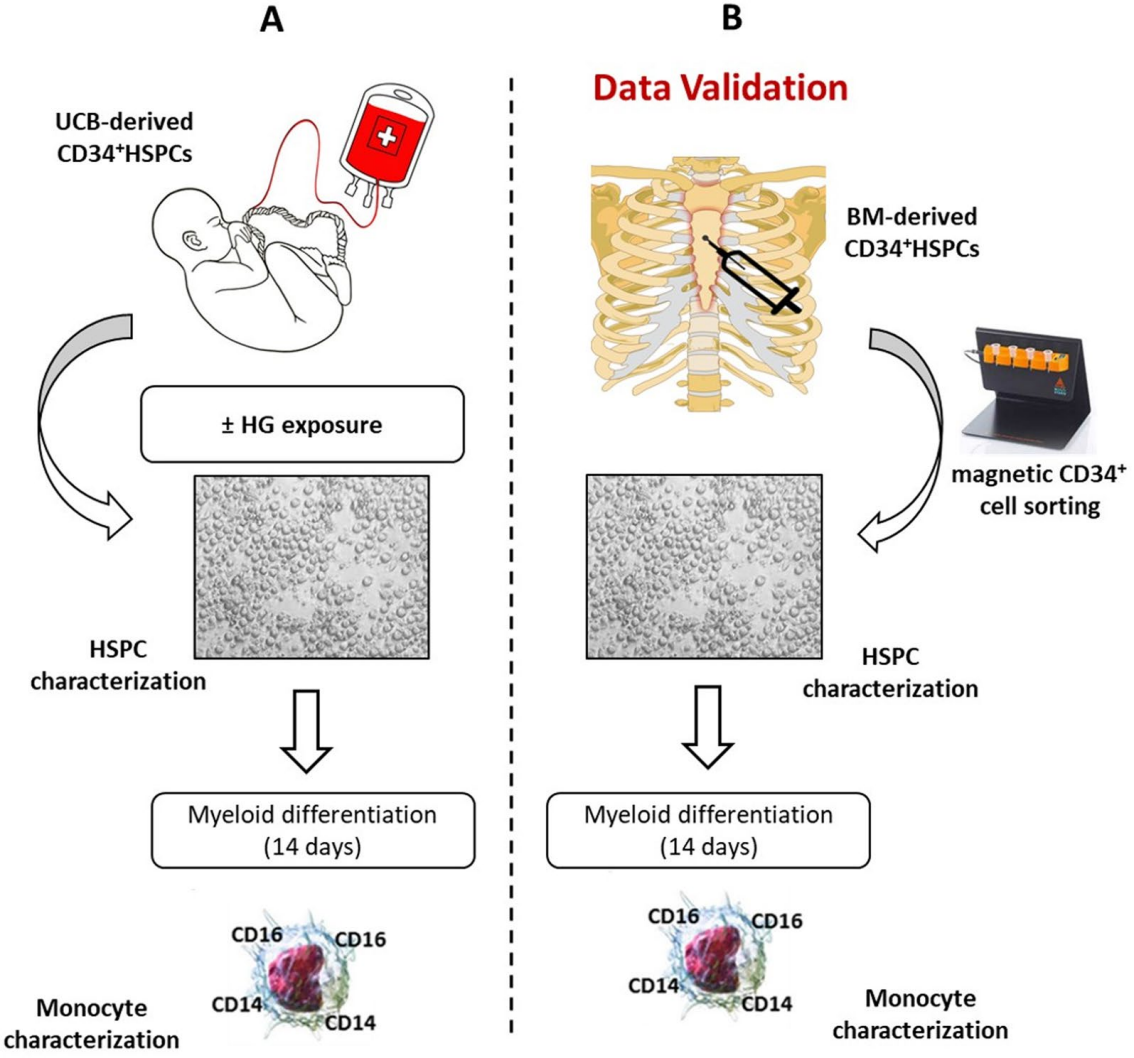
Schematic representation of the study. **A** UCB-derived CD34^+^ were expanded in HG conditions for 20 days. Afterward, CD34^+^HSPCs were phenotypically and epigenetically characterized before and after monocyte differentiation. The results were finally validated in BM-derived CD34^+^HSPCs from CAD and CAD-T2DM patients (**B**). UCB, umbilical cord blood; BM, bone marrow; HSPCs, hematopoietic stem/progenitor cells; NG, normal-glucose; HG, high-glucose

In the present study, we investigated the epigenetic mechanisms potentially contributing to hematopoiesis alteration in diabetic patients. To avoid confounding effects related to aging or other risk factors, we first evaluated the effects of high-glucose (HG) stimulation on epigenetically naïve umbilical cord blood (UCB)-derived CD34^+^ HSPCs. This in vitro approach allowed us to unequivocally identify those epigenetic modifications as a direct consequence of HG exposure. In addition, cells were recovered in normoglycemic conditions (NG) for three and ten days, to mimic in vitro metabolic memory, as previously described [13]. Then, after phenotypical, molecular, and epigenetic characterization, the cells were differentiated in vitro into the myeloid lineage and further analyzed. Finally, the findings of our in vitro model were validated in CD34^+^ HSPCs isolated from the sternal BM biopsies of coronary artery disease (CAD) patients undergoing bypass graft surgery with and without T2DM (Fig. 1).

### UCB CD34^+^ HSPC expansion

UCB-derived CD34^+^ HSPCs (Lonza; 2C-101A) were expanded up to 20 days in Stem Span (StemCell Technologies). The media was supplemented with 20 ng/ml of interleukin-6 (IL-6), 20 ng/ml of interleukin-3 (IL-3), 50 ng/ml of FMS-like tyrosine kinase 3 (FLT-3) and 50 ng/ml of Stem Cell Factor (SCF) (Peprotech) in hyperglycemic (30 mmol/L of glucose; HG) and normoglycemic (5 mmol/L of glucose plus 25 mmol/L of mannitol; NG) conditions. To reproduce the metabolic memory phenomenon in vitro, HG‐CD34^+^ stem cells were returned to physiological glucose conditions (5 mmol/L) for 3 days (exHG‐CD34^+^), as previously described [13].

### Study participants

All experiments have been carried out upon approval of local ethics committees (No. 2015/ST/232 and R196/14—CCM 205). Informed written consent was obtained from all patients before BM harvesting. CAD (n = 15) and CAD-T2DM (n = 17) subjects have been selected by stringent matching of age, pharmacological treatments, and major risk factors. The clinical characteristics of patients are shown in Table 1. All study participants did not receive immunosuppressive drug administration before surgery.

**Table 1:** Clinical profile, cardiovascular risk factors and therapies characterizing the subgroup of CAD patients enrolled on the study. Patients have been categorized for the presence of T2DM as a comorbidity

	CAD	CAD-T2DM	*P value*
N	**15**	**17**	
AGE (years)	65.33 ± 2.41	66.88 ± 2.31	0.65
BMI (KG/m^2^)	26.86 ± 0.87	27.13 ± 0.79	0.82
Glycemia (mg/dL)	103.1 ± 3.27	137.5 ± 10.31	0.004**
LDL (mg/dL)	120.4 ± 11.52	78.40 ± 9.74	0.0095**
HDL (mg/dL)	48.93 ± 3.58	45.60 ± 3.29	0.49
Total cholesterol (mg/dL)	189.1 ± 12.38	150.0 ± 9.67	0.012*
Creatinin(mg/dL)	1.068 ± 0.1	1.096 ± 0.1	0.84
Hypertension	**11**	**15**	
Dyslipidemia	**11**	**15**	
Smoke	**7**	**6**	
Oral antidiabetic agents	**0**	**14**	
Insulin	**0**	**1**	
Oral antidiabetic agents + Insulin	**0**	**2**	
Antihypertensive drugs	**12**	**15**	
Lipid-lowering drugs	**7**	**13**	

CAD ± T2DM, coronary artery disease with or without diabetes mellitus; BMI, body mass index; HDL, high-density lipoprotein; LDL, low-density lipoprotein. *P<0.05; **P<0.01 vs CAD

### Sternal MB biopsy and CD34^+^ stem cell isolation

Three mL of sternal blood from the BM of 32 patients with CAD ± T2DM was obtained by needle aspiration. Aspirates were resuspended in PBS and EDTA. Mononuclear fraction and CD34^+^ stem cells were isolated as previously described [18]. Based on the number of isolated cells, (from 2 × 10^5^ to 5 × 10^5^ cells/sample), CD34^+^ HSPCs were used for qPCR and/or differentiation and ChIP experiments.

### Statistical analysis

Results are given as mean SEM. All experiments were performed at least in triplicate unless stated otherwise. The data were tested for normality by using the Shapiro–Wilk normality test. Differences between data were evaluated by paired or unpaired Student t-test (2-group comparisons), 1-way, 2-way repeated-measures ANOVA followed by the post-hoc Dunnet multiple comparison test, as appropriate. A value of P ≤ 0.05 was considered significant. All statistical analysis was performed using GraphPad Prism software (GraphPad Software Inc.).

## Results

### High glucose exposure promotes a senescent-associate secretory phenotype in umbilical cord blood-derived HSPCs

**Fig. 2:**
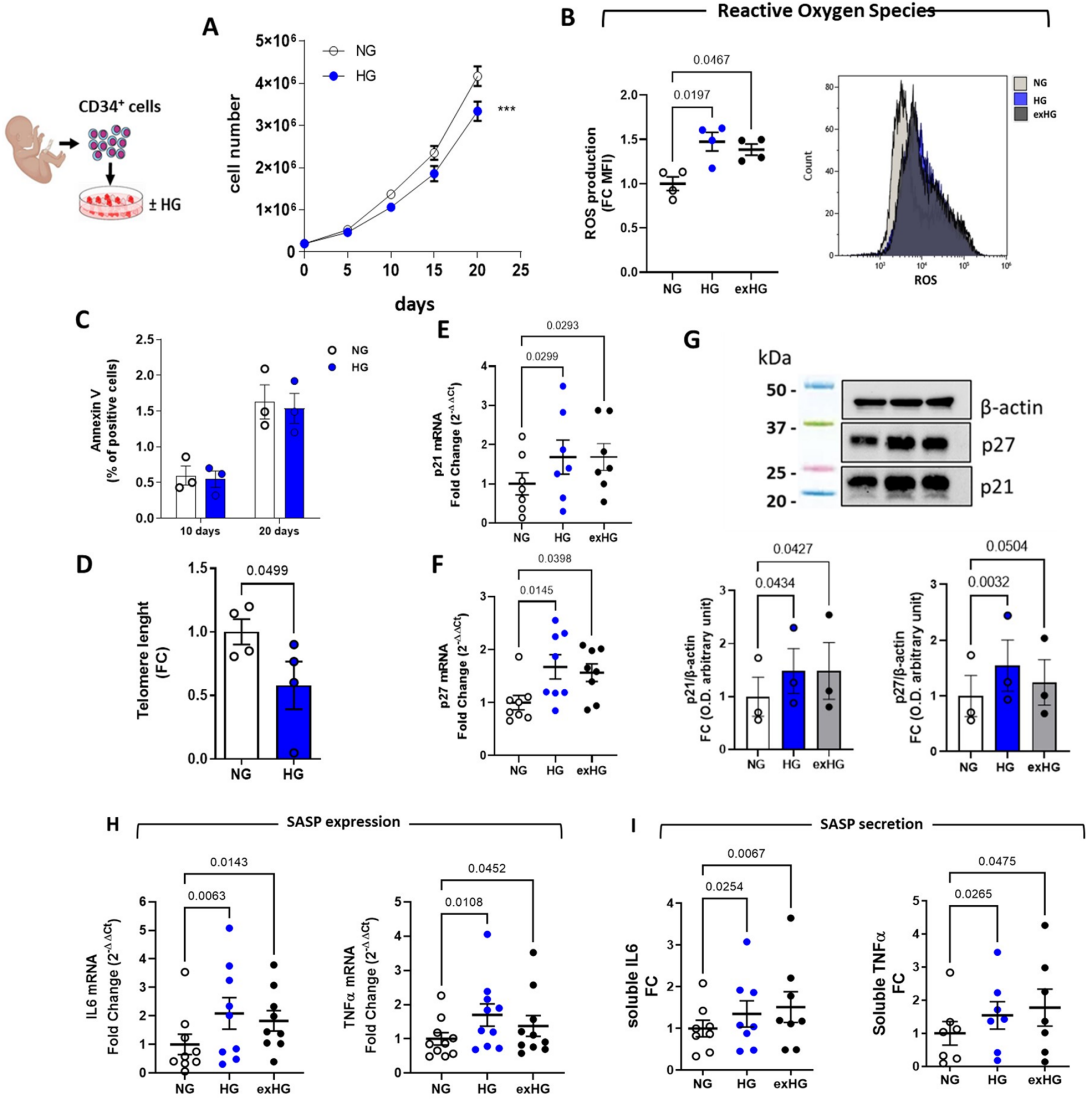
Effect of HG on CD34 + HSPC phenotype. **A** Proliferation curve of CD34^+^ cells exposed to HG condition (30 mmol/L; n = 5; ***P < 0.001; 2-way ANOVA). **B** and **C** flow cytometric quantification of ROS production and AnnexinV staining in HG-treated cells respectively. Data are reported as Fold-Change (FC) of Mean fluorescence intensity (MFI) over NG (B) (1-way ANOVA) and % of positive cells (**C**). **D** qPCR quantification of telomere length, data are expressed as FC (2^−ΔΔCt^; NG vs HG; paired t-test). **E** through **H** p21, p27, IL6 and TNFα expression analysis by qPCR and (**G**) Western blot (WB). Blot image is representative of 3 independent experiments. Data are represented as FC over NG (2^−ΔΔCt^ for qPCR; 1-way ANOVA). **I** secretion levels in culture media of IL6 and TNFα from HG and NG-CD34^+^ cells. Data are represented as FC over NG (1-way ANOVA)

We previously demonstrated that CD34^+^ HSPCs showed signs of metabolic exhaustion after 20-day of chronic HG exposure which was characterized by decreased proliferation rate and increased ROS production along with reduced expression of the antioxidant enzyme genes CAT and MnSOD [18]. Here, we have reproduced the same results to assess whether the observed impairment in cell proliferation was dependent on ROS-induced apoptotic mechanisms (Fig. 2A and B). The analysis by flow cytometry of Annexin V showed no differences between HG-CD34^+^ and NG-CD34^+^ cell populations, after both 10 and 20 days (Fig. 2C). Conversely, increased mitochondrial ROS production after 20 days of HG exposure in CD34^+^ cells were associated with a significant shortening of telomere length (Fig. 2D) and upregulation of cyclin-dependent kinase inhibitors genes (CDKi) p21 and p27 (Fig. 2E–G). In conjunction, the cells exhibited a pro-inflammatory phenotype characterized by upregulation and release into the supernatant of the pro-inflammatory cytokines, namely IL6 and TNFα (Fig. 2H and I). These findings, describing senescent and viable cells undergoing extensive changes in inflammatory cytokine expression and secretion, are consistent with the acquisition of a senescence-associated secretory phenotype (SASP) [19]. Noteworthy, upon restoration of NG conditions, CD34^+^ cells (exHG) persistently displayed an altered oxidative state that was associated with the upregulation, even after 10 days (Additional file 1: Fig. S1), of CDKi and proinflammatory genes, providing the first evidence of hyperglycemic memory of SASP in HSPCs (Fig. 2B and E–I).

### Exposure of HSPCs to high glucose alters histone methylation on NFkB-p65 promoter

**Fig. 3:**
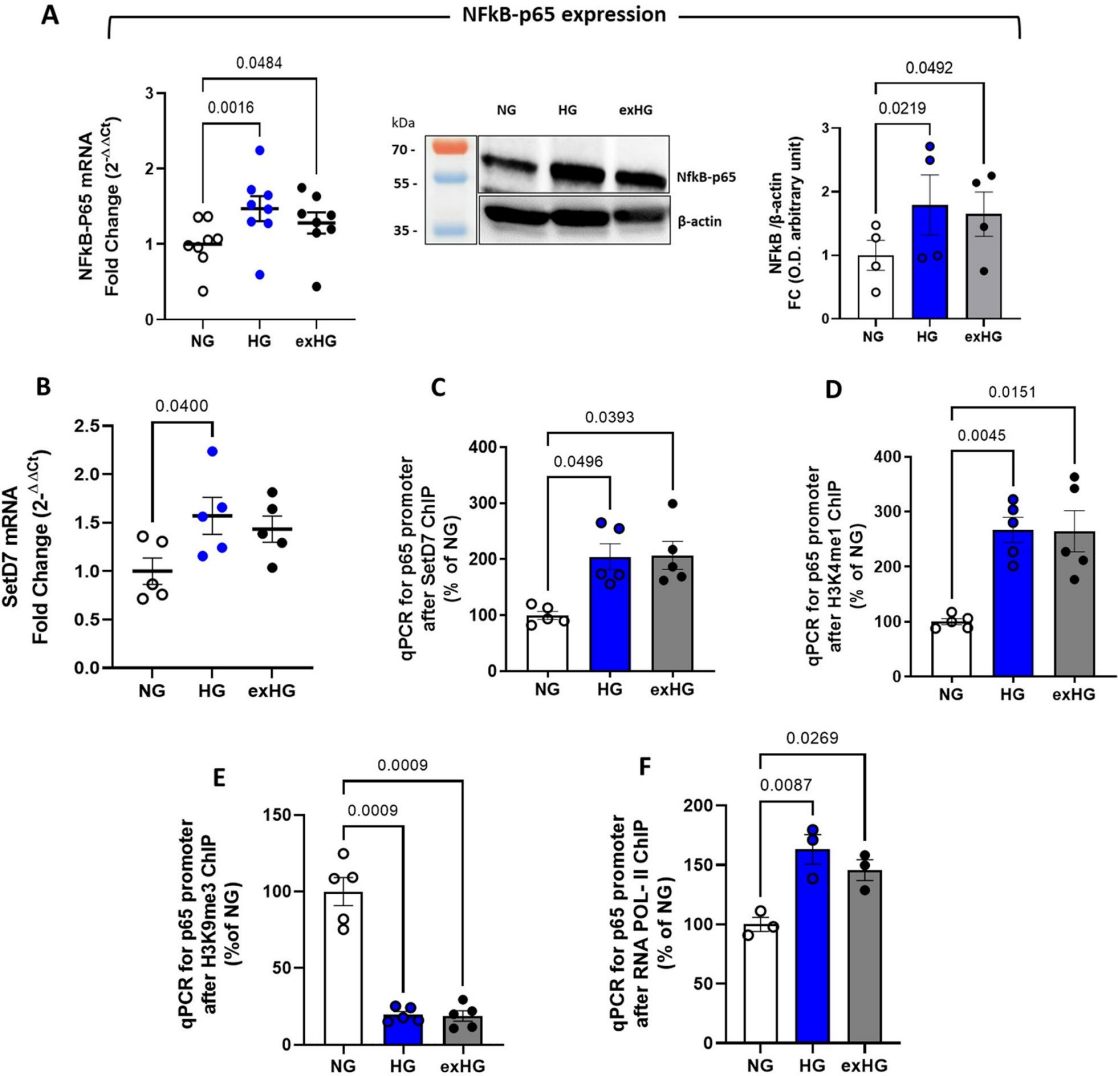
NFkB-p65 upregulation correlates with a more open chromatin conformation in HG-CD34 + cells. **A** evaluation of NFkB-p65 expression in NG-, HG-, and exHG-CD34 + cells by qPCR and WB analysis. Blot image is representative of at least 3 independent experiments. qPCR data are expressed as FC over NG (2^−ΔΔCt^; 1-way ANOVA). **B** qPCR quantification of SETD7 gene; the data are represented as FC over NG (2^−ΔΔCt^; 1-way ANOVA). **C** through **F**, ChIP of the p65 promoter by SETD7, H3K4me1, H3K9me3 and RNA POL II antibody in NG-,HG-, and exHG-CD34^+^ cells. The data, after input normalization, are expressed as a percentage of NG (1-way ANOVA). ChIP indicates chromatin immunoprecipitation; SETD7, SET (Su(var)3–9, Enhancer-of-zeste and Trithorax) domain containing lysine methyltransferase 7; H3K4me1, aminomethylation on lysine 4 of histone H3; H3K9me3, trimethylation on lysine 9 of histone H3; RNA POL II, RNA polymerase II

We next investigated the mechanistic pathway leading to SASP changes in HSPCs. Since NFκB-p65 is a transcription factor for numerous pro-inflammatory cytokines and the main inducer of SASP [20], we assessed its basal expression in NG- and HG-CD34^+^ cells. As shown in Fig. 3A, HG-CD34^+^ displayed a significant NFκB-p65 upregulation in terms of mRNA and protein when compared with osmotic control cells (NG-CD34^+^). We previously demonstrated a critical role for the SETD7 histone methyltransferase in H3K4me1-dependent upregulation of the NFkB-p65 subunit in T2DM [21]. Here, we tested whether SETD7-induced chromatin changes were also involved in the establishment of the SASP profile in HG-CD34^+^ cells. Real-time PCR analysis revealed a significant upregulation of the SETD7 gene in HG cells (Fig. 3B). Moreover, chromatin immunoprecipitation analysis displayed a sustained enrichment of SETD7 methyltransferase and H3K4me1 modification at the level of NFkB-p65 promoter in response to glucose (Fig. 3C and D). Interestingly, besides the accumulation of the gene-activating H3K4me1 mark, the p65 promoter also displayed a sustained reduction of the repressive H3K9me3 mark, indicating that the mutually antagonistic epigenetic modifications concurred with p65 gene activation (Fig. 3E). Finally, to determine whether these specific histone modifications correlated with increased p65 gene transcription, we further immunoprecipitated chromatin with antibodies against RNA polymerase II. The analysis confirmed higher RNA polymerase II engagement within the coding region of the p65 gene in HG-CD34^+^ cells (Fig. 3F). Of interest, we observed that HG-induced chromatin modifications persisted despite restoration of normoglycemia (exHG).

### High glucose epigenetically modulates the activation of NFkB-p65 transcription factor

**Fig. 4:**
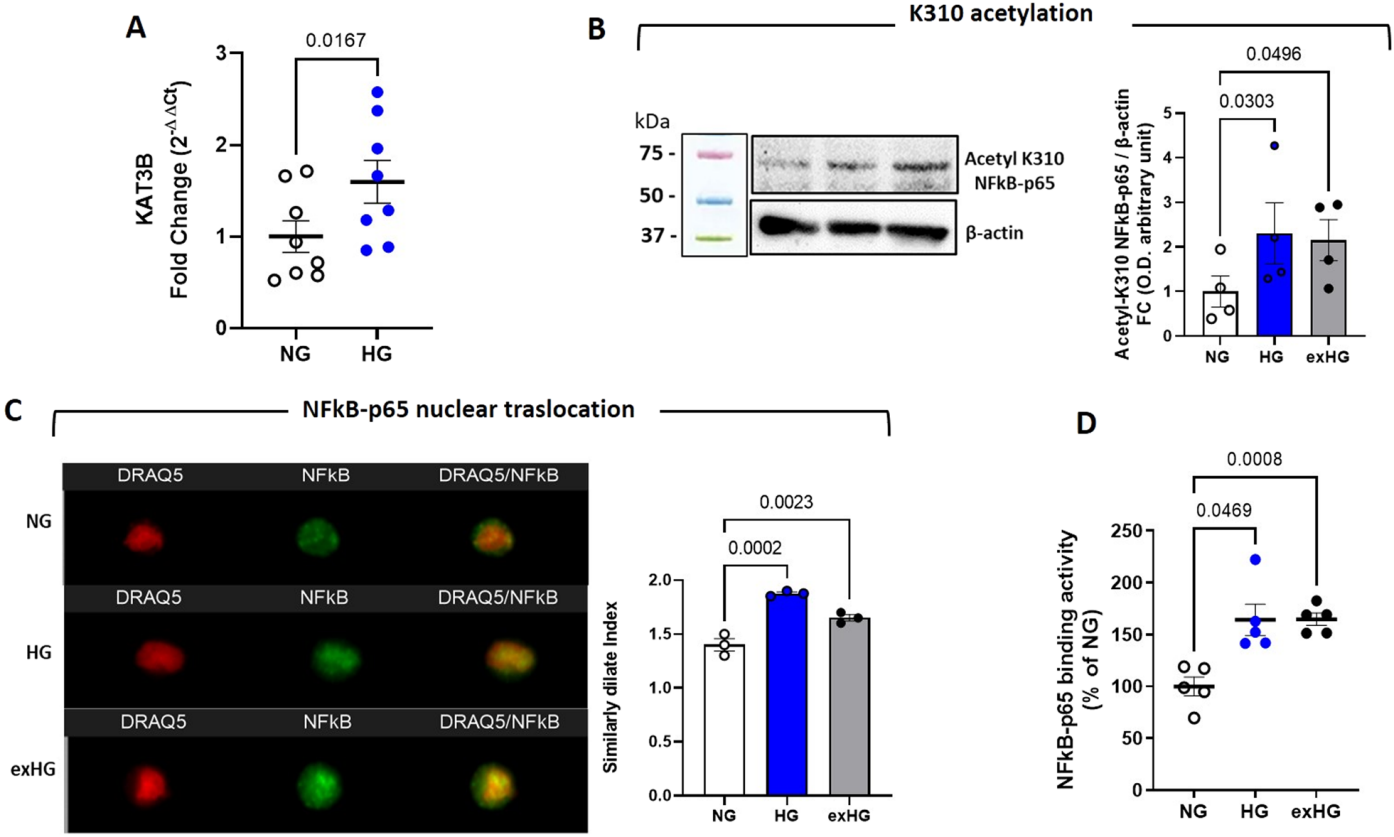
HG exposure modulates the activation of NFkB-p65 transcription factor by epigenetic mechanism. **A** qPCR quantification of KAT3B gene; the data are represented as FC over NG (2-^ΔΔCt^; NG vs HG; paired t-test). **B** Analysis of post-translational modification at lysine 310 (K310) by WB of p65 in HG and exHG-CD34^+^ cells. Blot image is representative of at least 3 independent experiments. **C** Nuclear compartmentalization analysis of NFkB-p65 in HG and exHG-CD34^+^ cells after DRAQ5™ and NFkB-p65 antibody staining by Amnis ImageStream^®^X Mk II. The merge fluorescence of DRAQ5/NFkB-p65 is expressed as Similarly Dilate Index. **D** Binding activity of NFkB-p65 in NG, HG, and exHG-CD34^+^ cells. Data are expressed as percentage of NG (1-way ANOVA). KAT3B, Lysine acetyltransferases (KATs), p300

Acetylation of specific lysine residue has been shown to regulate NFκB-p65 activation or deactivation. In particular, acetylation at lysine 310 (K310) by p300/KAT3B, a histone acetyltransferase, is critical for the full transcriptional activity of p65 [22]. Based on these findings, we extended our studies to elucidate epigenetic regulation of NFkB-p65 activity in a hyperglycemic context. We evaluated the expression of p300/KAT3B in HG-CD34^+^ cells. The data showed a significant upregulation of the p300/KAT3B gene along with increased acetylation at K310 of NFκB-p65 after HG exposure (Fig. 4A and B). Notably, consistent with enhanced acetylation at K310, Image Stream analysis revealed persistent nuclear confinement of NFkB-p65 that was associated with increased DNA binding activity in HG cells (Fig. 4C and D). Intriguingly, both post-translational modifications (PTM) at K310 and nuclear activity of NFkB-p65 persisted despite NG restoration.

### Myeloid differentiation of HSPCs exposed to high glucose promotes the generation of inflammatory monocyte subpopulation

**Fig. 5:**
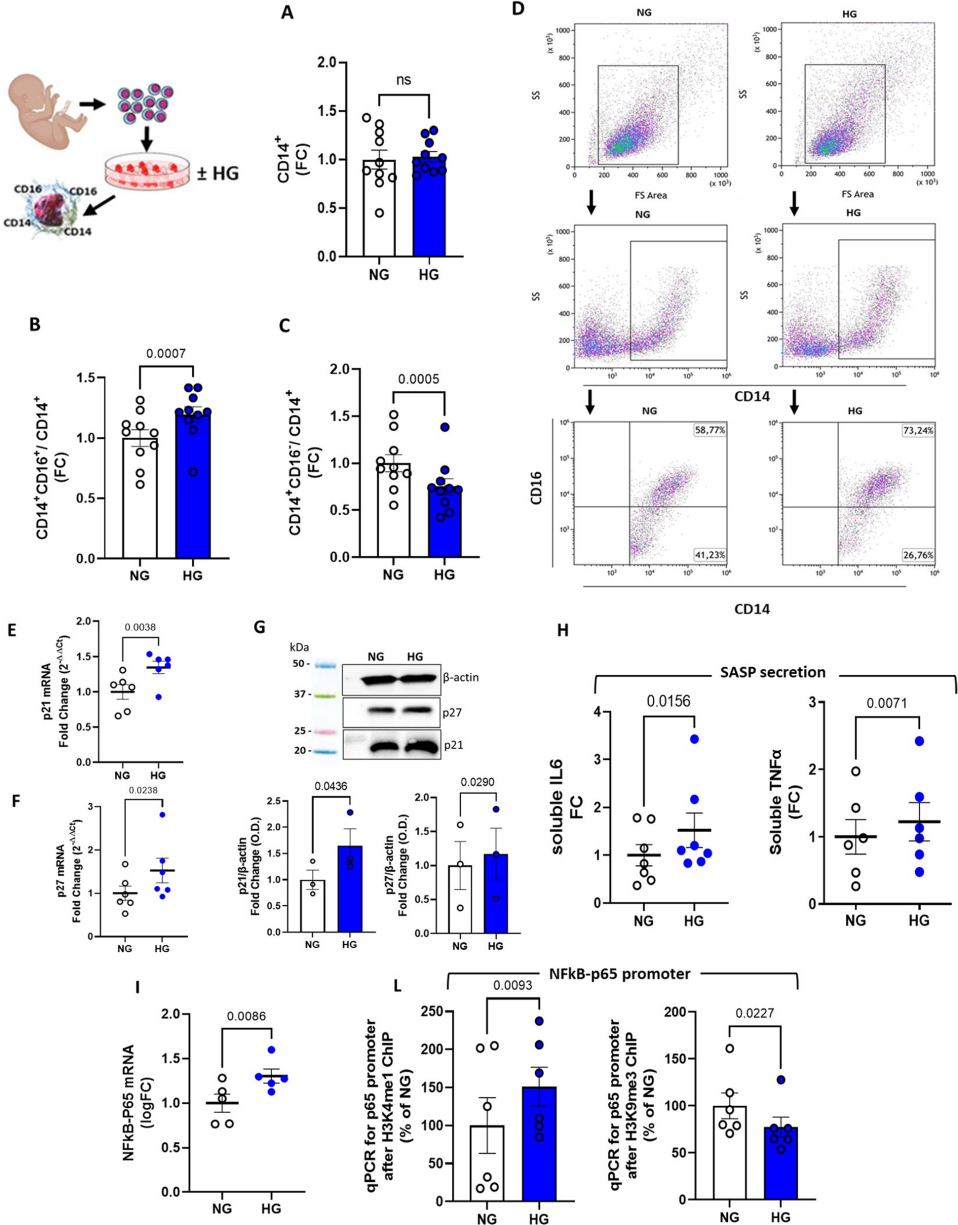
Phenotypical characterization of HG-CD34^+^- derived monocytes. **A** analysis of the total monocytes (CD14^+^) generated from HG-CD34^+^ cells after myeloid differentiation and (**B**) of their intermediate (CD14^hi^CD16^+^) and (**C **) classical (CD14^hi^CD16) subpopulations by flow cytometry. The data are expressed as FC (NG vs HG; paired t-test). **D** Flow cytometric dot blot analysis of a representative differentiation experiment. **E** through **G**, p21 and p27 gene expression analysis by qPCR (2^−ΔΔCt^) and WB. The data are expressed as FC (NG vs HG; paired t-test). **H** quantification of proinflammatory cytokines released in the medium after LPS stimulation. The data are expressed as FC (NG vs HG; paired t-test). **I** p65 gene expression analysis by qPCR (2^−ΔΔCt^) and (**L**) quantification of H3K4me1, H3K9me3 modifications on its promoter by ChIP assay. The data are expressed as FC (NG vs HG; paired t-test)

Monocytes and monocyte-derived macrophages play a pivotal role in the development of atherosclerotic cardiovascular disease (CVD) [23]. Human monocytes, defined by the CD14 marker expression, encompass a subset of monocytes with highly pro-inflammatory properties, namely intermediate (CD14^hi^CD16^+^) and non-classical (CD14^low^CD16^+^) monocytes, distinguished from “classical” monocytes by concomitant expression of the CD16 marker [24]. The percentage of these two subsets among classical CD14^+^ monocytes is reported to increase in various inflammatory diseases, including CAD and diabetes [23, 25, 26]. To test whether the epigenetic priming promoted by HG exposure in HSCs was itself sufficient for propelling the generation of proinflammatory monocyte subsets, we differentiated the cells toward myeloid lineage [27]. As shown in Fig. 5A, HG exposure did not affect the generation of total CD14^+^ cells, nonetheless, subset analysis by CD16 marker staining revealed significant alterations in monocyte composition deriving from HG-CD34^+^ cells, characterized by a significant accumulation of aggressive intermediate (CD14^hi^CD16^+^) monocytes at the expense of the classic ones (CD14^hi^CD16^−^) (Fig. 5B and C).

### Monocytes derived from HG-treated HSPCs exhibit a similar epigenetic pattern and display SASP features

Our study hypothesizes that epigenetic changes in HSPCs, as a result of hyperglycemic stress, are responsible for the commitment of differentiated immune effector cells with an inflammatory phenotype. In particular, we posit that HSPC-derived immune cells acquire maladaptive epigenetic signals that are perpetuated despite glucose normalization and are critically involved in the initiation and maintenance of systemic and vascular inflammation in diabetes. Hence, we analyzed the phenotype of the myeloid progeny derived from HG-treated CD34^+^ cells. Noteworthy, HG exposure of HSPCs elicited the generation of monocytes with SASP profile that were characterized, as their ancestors, by increased CDKi p21 and p27 gene expression (Fig. 5E–G) and release, after LPS stimulation, of proinflammatory IL6 and TNFα cytokines (Fig. 5H). Interestingly, differently from HG-treated HSPCs, HG-CD34^+^  -derived monocytes also showed a significant upregulation of ILα and Ilβ genes after LPS stimulation when compared to their NG counterparts (Additional file 1: Fig. S2 and S3). To note, the expression of the NFkB-p65 gene, a hallmark of SASP, was still upregulated and p65 promoter analysis confirmed the persistence of the H3K4me1/H3K9me3 histone modification pattern (Fig. 5I and L). These data suggest that chromatin changes initiated by HG can be transmitted across cell generations thus leading to altered transcriptional programs and phenotypic changes fostering inflammation and senescence.

### BM-derived HSPCs from T2DM patients express SASP genes

**Fig. 6:**
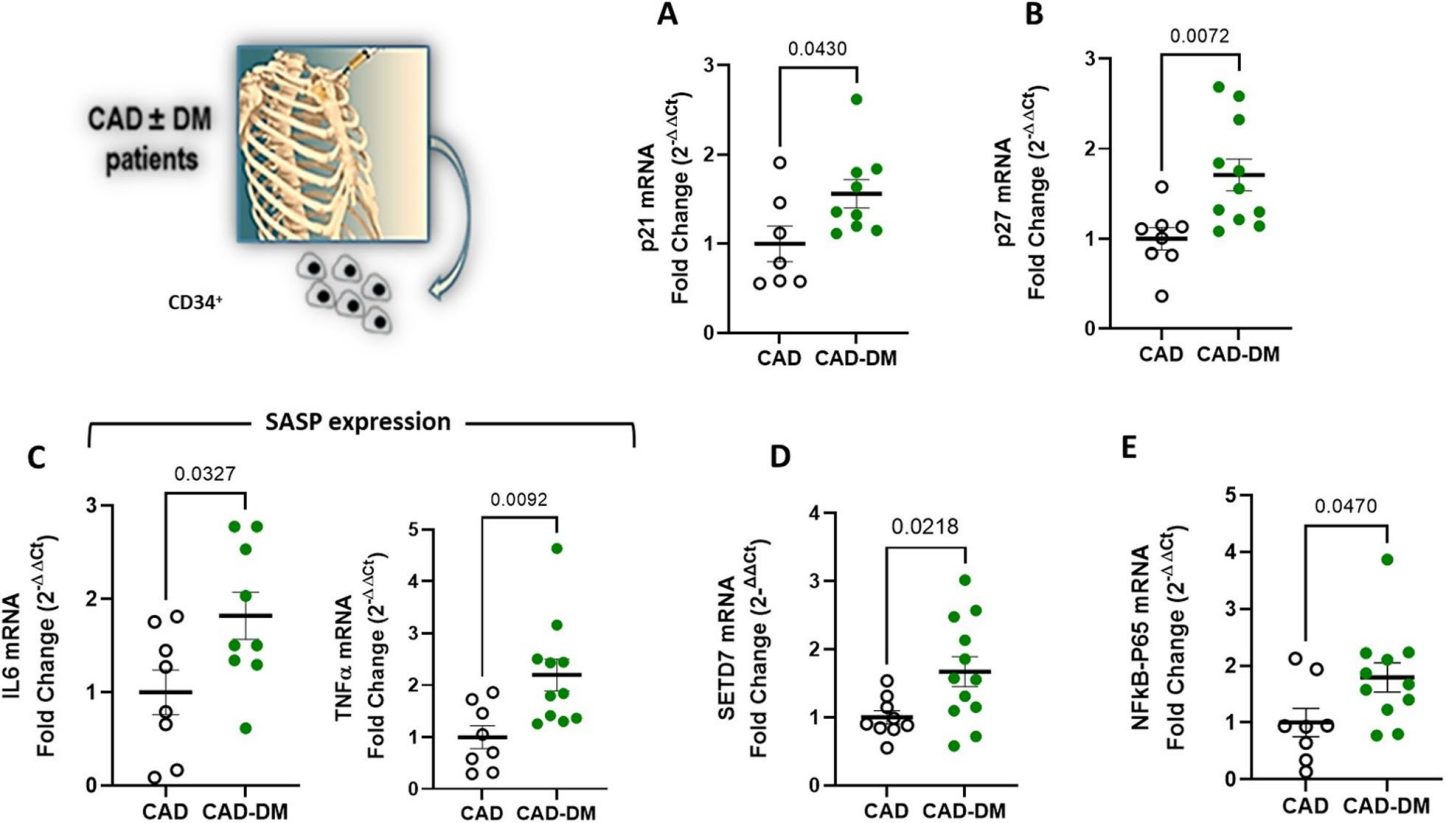
SASP analysis of CD34^+^ HPSCs isolated from sternal bone marrow biopsy of CAD ± T2DM patients.** A **through** E** p21, p27, IL6, TNFα, SETD7 and NFkB-p65 gene expression analysis by qPCR. Data are represented as FC over NG (2^−ΔΔCt^; CAD vs CAD-T2DM; unpaired t-test)

To explore the potential clinical relevance of our observations, we isolated CD34^+^HSPCs from the sternal BM biopsies of CAD patients with and without T2DM who underwent elective bypass surgery. Consistent with our in vitro model of hyperglycemia, CD34^+^ cells from CAD-T2DM patients displayed, a significant upregulation of CDKi p21 and p27 genes (Fig. 6A and B) as well as upregulation of proinflammatory genes (IL6 and TNFα), and their transcription factor NFkB-p65 (Fig. 6C and E). Interestingly, SETD7 gene also resulted upregulated (Fig. 6D), reaffirming the contribution of epigenetic mechanisms in the senescent and proinflammatory setting of CAD-T2DM HSPCs.

### In vitro-differentiated HSPCs from T2DM patients generate proinflammatory monocyte sub-populations

**Fig. 7:**
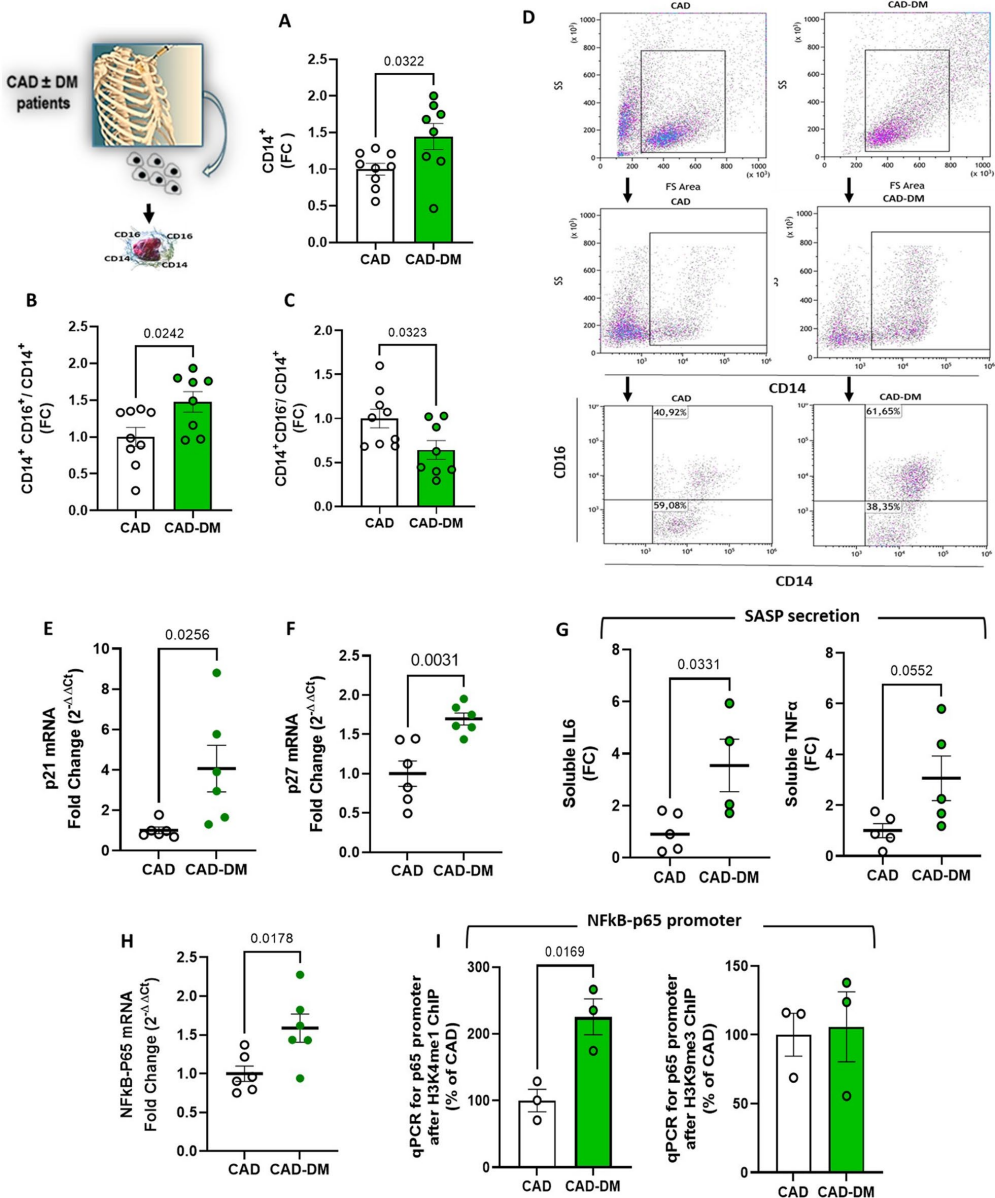
Phenotypical characterization of CAD ± T2DM-CD34^+^ HSPC-derived monocytes. **A** analysis of the total monocytes (CD14^+^) generated in vitro from CAD ± T2DM-CD34^+^ cells after myeloid differentiation and (**B**) of their intermediate (CD14^hi^CD16^+^) and (**C**) classical (CD14^hi^CD16) subpopulations by flow cytometry. The data are expressed as FC (CAD vs CAD-T2DM; unpaired t-test). **D** Flow cytometric dot blot analysis of a representative differentiation experiment. **E** and **F** p21 and p27 gene expression analysis by qPCR (2^−ΔΔCt^) and **G** quantification of proinflammatory cytokines released in the medium after LPS stimulation. The data are expressed as FC (CAD vs CAD-T2DM; unpaired t-test). **H** p65 gene expression analysis by qPCR (2^−ΔΔCt^) and **I** quantification of H3K4me1, H3K9me3 modifications on the promoter by ChIP assay. The data are expressed as FC (CAD vs CAD-T2DM; unpaired t-test)

To assess whether the diabetic BM environment was itself sufficient to promote hematopoietic alterations, CAD ± T2DM CD34^+^ cells were differentiated in vitro as previously described. As shown in Fig. 7A, the myeloid commitment of CAD-T2DM CD34^+^ cells resulted in a significantly higher generation of monocytes when compared with CAD. Interestingly this finding was in line with the clinical literature supporting the notion that myelopoiesis is increased in diabetic patients [3]. Noteworthy, cell population analysis revealed, like our in vitro model of hyperglycemia, a significant accumulation of intermediate monocytes (CD14^hi^CD16^+^) and reduction of the classical population (CD14^hi^CD16^−^) in CAD-T2DM patients when compared with CAD (Fig. 7B and C).

### CAD-T2DM-derived monocytes display SASP profile and gene-activating H3K4me1 histone mark at the NFkB-p65 promoter

We then characterized the monocytes generated from patient-derived HSPCs to assess whether the myeloid progeny retained the phenotype of the ancestors. In line with our in vitro model of hyperglycemia, at the end of differentiation, CAD-T2DM-derived monocytes still showed, as their precursors, high expression levels of CDKi p21 and p27 genes, and release, after LPS stimulation, of proinflammatory IL6 and TNFα cytokines (Fig. 7E–G). Additionally, the expression of SASP genes associated with a persistent upregulation of the NFkB-p65 transcription factor (Fig. 7H). These findings prompted us to evaluate whether, like our in vitro model of diabetes, a similar pro-transcriptional histone modification pattern was present at the level of p65 promoter. Consistently, chromatin immunoprecipitation of CAD-T2DM-derived monocytes showed a significant accumulation of activating H3K4me1 epigenetic modification, whereas no changes were observed in the level of H3K9me3 (Fig. 7I), suggesting a non-complete recapitulation of the bone marrow environment.

## Discussion

DM is accompanied by a state of chronic low-grade inflammation that contributes to the development of various complications, including CVD, the most common cause of death and disability in this class of patients [28]. Hyperglycemia is known to exacerbate CVD, however intensive glucose lowering approaches, a cornerstone of DM treatment, were not associated with a commensurate reduction of CV risk [29–32]. These paradoxical clinical findings were recently explained with the “metabolic memory” phenomenon, according to which previous episodes of hyperglycemia are able to induce maladaptive chromatin changes and transcriptional programs which are not restored even after restoration of normoglycemia thus leading to persistent cellular damage [33–35]. In this regard, the landmark Diabetes Control and Complications Trial (DCCT) and the follow-up study, Epidemiology of Diabetes Interventions and Complications (EDIC), demonstrated not only that intensive glycemic control in subjects with type 1 diabetes reduced the risk of microvascular complications but also that episodes of poor glycemic control can lead many years later to the long-term complications of diabetes [36, 37]. A better understanding of the mechanisms underpinning hyperglycemic memory may contribute to the development of innovative strategies to tackle vascular inflammation in patients with diabetes. Numerous clinical studies describe profound alterations of hematopoiesis in patients with DM, in which increased myelopoiesis and hyperglycemia-induced trained immunity appear to drive inflammatory processes and vascular damage [38–42]. Furthermore, a growing number of studies in animal models of DM suggest that diabetes/hyperglycemia may reprogram BM cells and promote a proinflammatory state by epigenetic modifications that can be eventually transmitted to their progeny [7, 11, 43–45]. Previous studies suggest DM-induced epigenetic modifications can foster maladaptive inflammatory alterations in differentiated immune cells. To this regard, evidence of hyperglycemia-induced trained immunity was demonstrated in human PBMCs from patients with T2DM [11]. However, even if it could be inferred that the hyperglycaemia-induced trained immunity observed in human PBMCs from T2DM patients could be initiated at the level of BM cells, no previous work has demonstrated this hypothesis in HSPCs from BM of patients [46, 47]. By leveraging an in vitro DM model based on cord-blood derived CD34^+^ HSPCs, in the present study we provide pre-clinical evidence that HG exposure per se is sufficient to epigenetically promote a senescent and pro-inflammatory phenotype, resembling SASP. Interestingly enough, hyperglycemia-induced epigenetic changes and subsequent inflammatory/senescent signatures were shown to persist despite normalization of glucose levels in the cell media, suggesting the existence of hyperglycemic memory in this setting. Specifically, in line with our previous findings [18], chronic HG exposure of CD34^+^ HSPCs induced mitochondrial ROS production, recognized as the upstream event triggering SASP-related changes [48]. In this respect, HG-CD34^+^ cells displayed a reduced proliferation rate, telomere loss, and P21 and P27 gene upregulation associated with increased secretion of two of the major components of the pro-inflammatory SASP, the cytokines IL6 and TNFα. Finally, upregulation of the transcription factor NFkB-p65 confirmed the ROS-NFκB axis's involvement in the proinflammatory phenotype of HSPCs [14, 48]. NFkB is a ubiquitous transcription factor important in regulating many cytokines and chemokines, whose transcription is known to be activated by epigenetic mechanisms in T2DM [46, 49, 50]. In cellular studies, Paneni and co-authors demonstrated the involvement of the histone methyltransferase SETD7 in the epigenetic regulation of NFkB-dependent oxidative and inflammatory signaling in peripheral blood mononuclear cells (PBMCs) from patients with T2DM. His lab demonstrated that PBMCs derived from patients with T2DM showed upregulation of SETD7 and SETD7-dependent mono-methylation of histone 3 at lysine 4 on the NFkB p65 promoter [21]. Consistently, here we show that HG exposure of CD34^+^ HSPCs was itself sufficient for increasing the expression and recruitment of SETD7 at the level of p65 promoter, resulting in a significant enrichment of H3K4me1. Interestingly, the accumulation of the active histone mark H3K4me1, associated with reduced levels of the repressive H3K9me3 mark, mechanistically reaffirmed that histone marks with opposing functions fine tune gene expression in our setting [51]. Specifically, the imbalance between activating and repressing marks toward the former led to increased recruitment of RNA polymerase II on the p65 gene promoter with subsequent gene upregulation and inflammatory response. Noteworthy, we found that HG-treated CD34^+^HSPCs also exhibited a significant upregulation of the p300/KAT3B gene, a histone acetyltransferase involved in the K310 acetylation of p65 [22]. This site-specific p300-mediated acetylation of p65 is known to regulate transcriptional activity and specificity of NF-kB-target gene subsets [52]. Accordingly, HG-CD34^+^ showed increased acetylation in K310 of p65, which associated with its increased nuclear localization and DNA-binding. However, although acetylation at K310 is critical for the full transcriptional activity of p65, it does not affect DNA binding capacity suggesting that other PTMs could be also involved [52]. Notably, similar to the epigenetic modifications at the promoter level, both the PTM at K310 and NFκB-p65 transcriptional activity persisted in HG-CD34^+^ cells despite correction of the glucose concentration, providing the first evidence that “hyperglycaemic memory” also drives persistent cell signaling in BM-derived cells.

Besides histone modifications, a recent study showed that hyperglycemia associates with changes in DNA methylation of NF-kB gene promoter [53]. Of interest, treatment with GLP-1R agonists was able to restore promoter methylation thus suppressing NF-KB expression. In addition, GLP-1R stimulation by liraglutide, showed to protect CD34^+^ HSPCs from dysfunction induced by high glucose exposure [54]. These latter findings suggest that: (i) DNA methylation changes might also play a significant role in our setting; (ii) GLP-1R agonists could be useful in preventing hyperglycemia-induced trained immunity and monocyte inflammation. Future studies should address these important aspects.

Although our previous work demonstrated a significant increase in ROS levels in CD34 + cells [18], the role of oxidative stress in NF-kB activation and senescence of CD34 + cells remains elusive. Chronic treatment with the antioxidant N-acetyl cysteine (NAC) for 20 days resulted to be toxic in vitro and, hence, we could not test the impact of this strategy in rescuing cell senescence in our experimental setting. Alternative strategies can be considered to overcome this issue and include the generation of a cell line with constitutive overexpression of MnSOD to test the long-term antioxidant effects on cellular senescence. Follow-up work from our lab will contribute to elucidate the biological link between hyperglycemia-induced ROS generation, inflammation and cellular senescence.

Finally, in vitro, myeloid differentiation of HG-CD34^+^ cells led to the generation of monocytes mainly composed of a more inflammatory intermediate subpopulation (CD14^hi^CD16^+^). Similarly to their ancestors, HG-CD34^+^-derived monocytes displayed a SASP profile in which NFkB-p65 gene upregulation was characterized by the same epigenetic modifications at the promoter level, suggesting the transfer of information to the daughter cells. Notably, the persistence of the pro-inflammatory epigenetic priming in the monocyte progeny was likely a determinant to elicit a greater response after LPS stimulation. The results of our in vitro model immediately prompted us to investigate whether similar mechanisms were at work in CD34^+^ HSPCs derived from the sternal BM of diabetic patients. Accordingly, BM-CD34^+^ HSPCs from T2DM patients showed, besides SETD7 upregulation, a SASP profile characterized by the overexpression of p21, p27, NFkB-p65, and NFkB target TNFα/IL-6 genes, demonstrating that T2DM is a powerful driver of organismal aging which also includes stem cells [55]. Noteworthy, CAD-T2DM-derived CD34^+^ HSPCs displayed, despite in vitro culture and differentiation, a “glucophenotype” characterized by myelopoietic and inflammatory features that were irrespective of the presence of CAD and of lipidemic profile of the patients [56]. These data suggest that additional epigenetic mechanisms and transcription factors, other than those reproduced in our limited in vitro model, are involved in the trained innate immunity of T2DM patients [57]. Finally, as we observed in HG-CD34^+^-derived monocytes, the epigenetic analysis of NFkB-p65 promoter in CAD-T2DM-derived monocytes revealed pro-transcriptional H3K4me1 mark accumulation that, following our hypothesis, was the faithful reflex of the “fixed” HSPC reprogramming promoted by T2DM in the BM. Until now, studies on HSPCs derived from human BM have generally been elusive due to the difficulty of obtaining samples and quantities suitable for experimentation outside the context of hematological disease. Although we acknowledge that both the in vitro model of diabetes and the myeloid differentiation protocol of CD34^+^ HSPCs cannot fully recapitulate the environment of the diabetic BM and that the small number of cells obtained from sternal BM biopsies may limit the validation of the study, overall these data provide experimental evidence for hyperglycaemia/diabetes-induced reprogramming of haemopoiesis in human CD34^+^ HSPCs.

An important aspect to be discussed is for “how long” persistent epigenetic changes can be observed after normoglycemia restoration. After longer periods of recovery in normoglycemia (up to 10 days) exHG-CD34^+^ cells still showed gene expression alteration (Additional file 1: Fig. S1). Of interest, we found that CD34^+^ HSPCs also displayed long-lasting epigenetic changes that were transmitted to daughter cells. In line with our results, El-Osta et al. showed that prolonged periods of normoglycemia restoration were associated with persistent upregulation of NFkB-p65 subunit and endothelial inflammation [14]. In patients with type 2 diabetes and HbA1c ≥ 7.5%, Costantino et al. found that 6 months of optimal glycemic control were not sufficient to reset epigenetic changes (DNA methylation and histone acetylation) in circulating PBMCs, suggesting a long-lasting effect of hyperglycemia on the epigenome [58]. In our study, we have obtained similar results when using UCB-derived CD34^+^ HSPCs and BM-derived CD34^+^ HSPCs from diabetic individuals (usually middle age-old individuals). Interestingly, although a direct comparison (young vs old) is lacking, these data suggest that age per se might not affect progenitor cell response to HG. Further work investigating age-dependent epigenetic signals is needed to draw solid conclusions on this relevant aspect.

In conclusion, by the use of an in vitro model further corroborated on BM patient-derived CD34^+^ HSPCs, we show for the first time that hyperglycemia induces marked epigenetic changes responsible for long-term proinflammatory priming of HSPCs, which, once transmitted to the cell progeny, contribute to persistent and pathogenic changes in immune cell function and composition even after the original stimulus (HG) was removed. This condition, known as trained immunity, has an important role in the development and progression of diabetic cardiovascular complications and could explain the lack of efficacy of conventional treatments in the management of the vascular risk and complications of diabetes, still merely focused on glucose reduction. Our findings have important implications for the management of diabetes-related atherosclerotic complications. We show that a pro-inflammatory phenotype of myeloid cells and BM precursors persist after normalization of blood glucose. The identification of specific epigenetic targets set the stage of mechanism-based strategies to prevent hyperglycemia-induced trained immunity. Recent work has shown that pharmacological inhibition of SETD7 by (R)PFI-2 is able to prevent cellular inflammation and oxidative stress in vitro [59]. Our study may set the stage for epigenetic therapies (i.e. SETD7-targeting approaches) aimed at preventing the activation of pathways (i.e. NFkB) driving trained immunity and pro-atherosclerotic features in diabetes. Hyperglycemia-induced trained immunity might also affect the defense mechanisms against pathogens and explain the increased risk of infections in diabetic patients.

### Supplementary Information

**Additional file 1: Figure S1.** **A** through **E**, analysis of p21, p27, IL6, TNFα and NFkB-p65 genes by qPCR in CD34^+^ HSPCs after 10 days of recovery in normoglycemic condition. Data are represented as FC over NG (2^-ΔΔCt^; 1-way ANOVA). **Figure S2.** **A** and **B** gene expression analysis by qPCR of IL1α and Ilβ in CD34^+^ HSPCs after 20 days HG exposure. Data are represented as FC over NG (2^-ΔΔCt^; 1-way ANOVA). **Figure S3.** **A** and **B** gene expression analysis by qPCR of IL1α and Ilβ in HG-CD34^+^ HSPC-derived monocytes after LPS stimulation. Data are represented as FC over NG (2^-ΔΔCt^; 1-way ANOVA).

**Additional file 2: Table S1.** Primer List for qPCR. **Table S2.** Antibody list.

**Additional file 3: ** Additional material & methods.

## Data Availability

Data are available upon request.
