# Supplementary material for: Persistent epigenetic signals propel a senescence-associated secretory phenotype and trained innate immunity in CD34+ hematopoietic stem cells from diabetic patients
Source: Cardiovasc Diabetol. 2024 Mar 29;23:107. doi: 10.1186/s12933-024-02195-1 (PMC10981360; doi:10.1186/s12933-024-02195-1)
Supplement: Supplementary file 1 — Additional file 1: Figure S1. A through E, analysis of p21, p27, IL6, TNFα and NFkB-p65 genes by qPCR in CD34+ HSPCs after 10 days of recovery in normoglycemic condition. Data are represented as FC over NG (2-ΔΔCt; 1-way ANOVA). Figure S2. A and B gene expression analysis by qPCR of IL1α and Ilβ in CD34+ HSPCs after 20 days HG exposure. Data are represented as FC over NG (2-ΔΔCt; 1-way ANOVA). Figure S3. A and B gene expression analysis by qPCR of IL1α and Ilβ in HG-CD34+ HSPC-derived monocytes after LPS stimulation. Data are represented as FC over NG (2-ΔΔCt; 1-way ANOVA). [file 12933_2024_2195_MOESM1_ESM.pptx]

## Slide 1
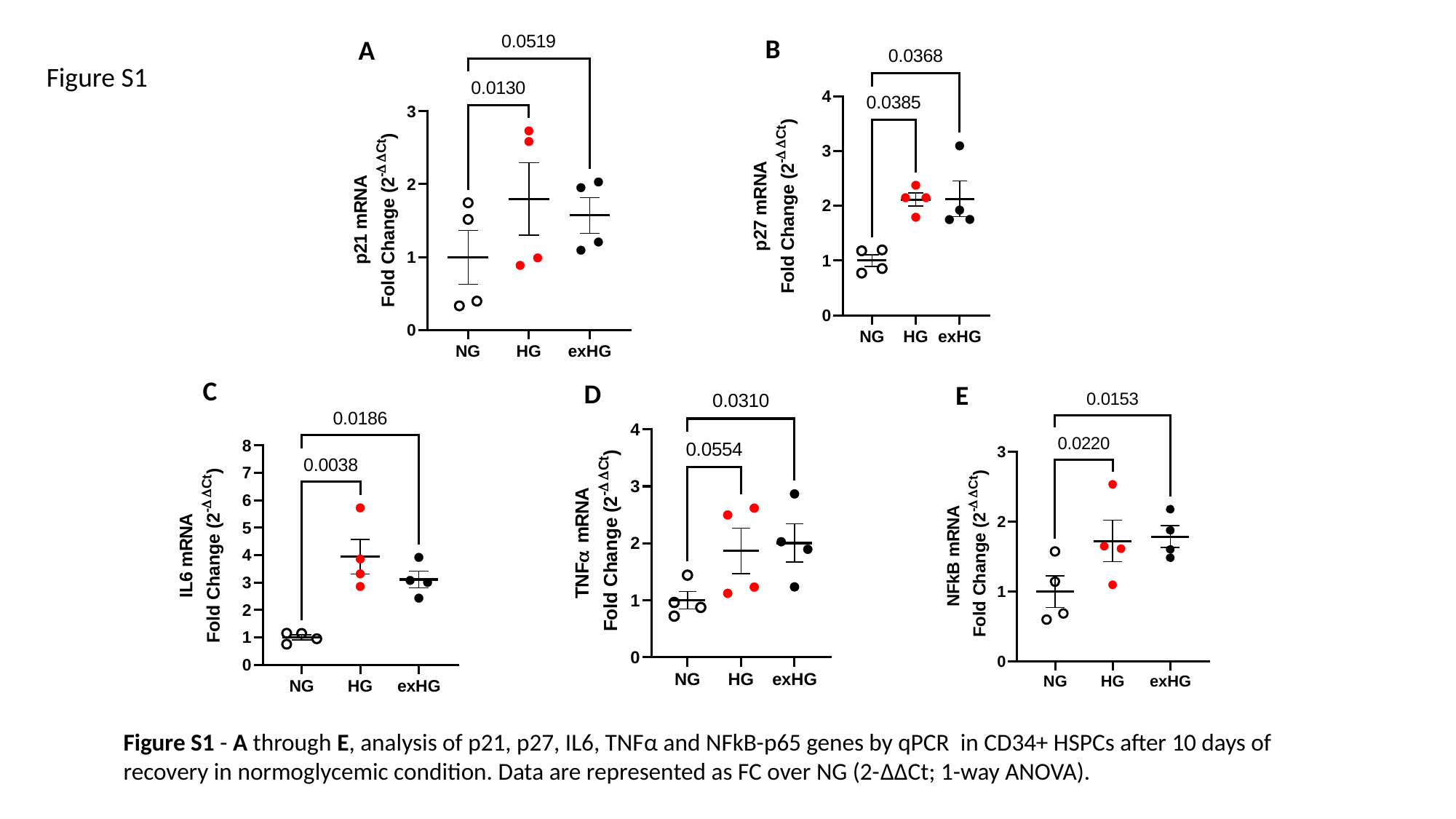

B
A
C
D
E
Figure S1
Figure S1 - A through E, analysis of p21, p27, IL6, TNFα and NFkB-p65 genes by qPCR in CD34+ HSPCs after 10 days of recovery in normoglycemic condition. Data are represented as FC over NG (2-ΔΔCt; 1-way ANOVA).

## Slide 2
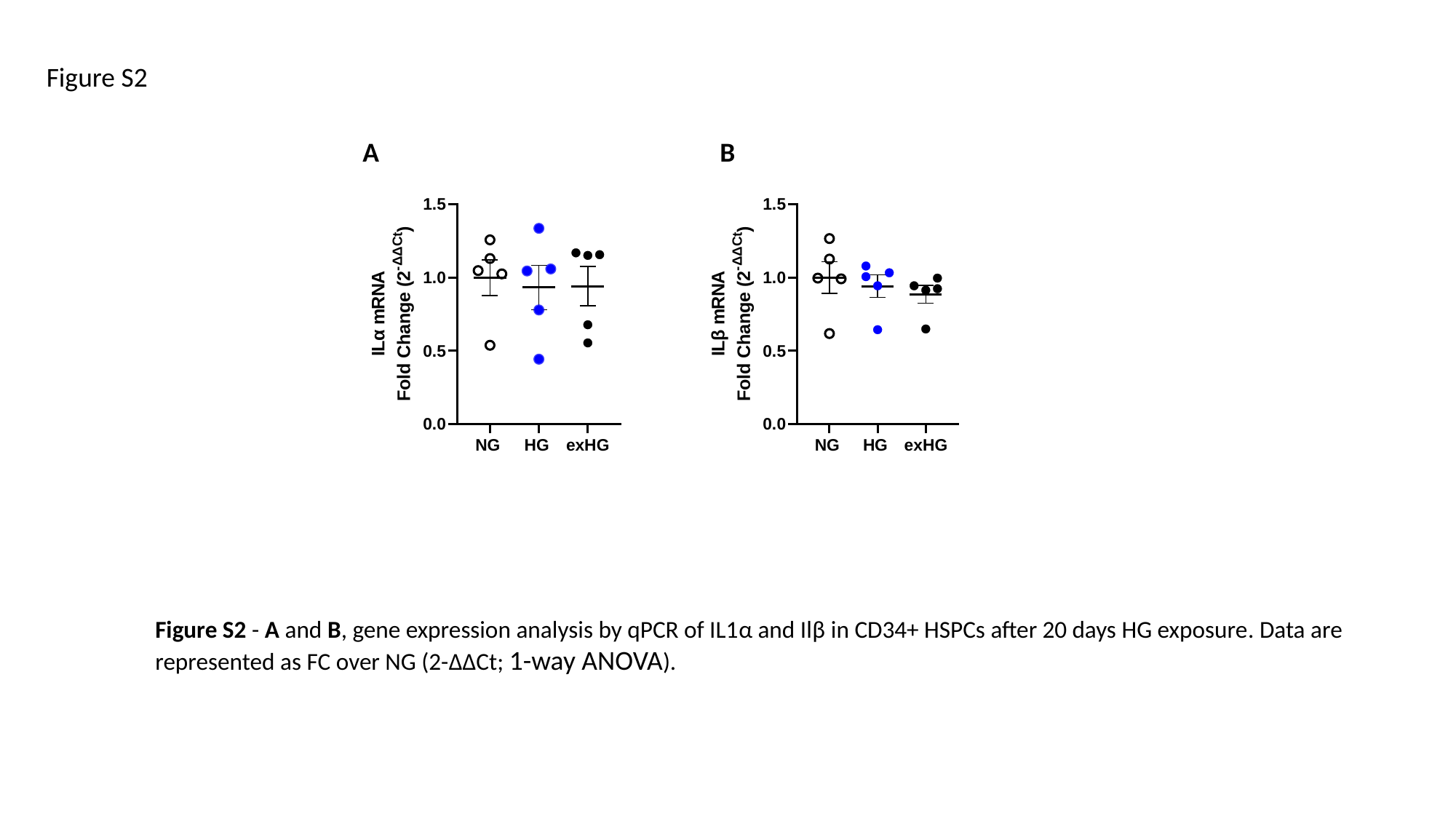

Figure S2
A
B
Figure S2 - A and B, gene expression analysis by qPCR of IL1α and Ilβ in CD34+ HSPCs after 20 days HG exposure. Data are represented as FC over NG (2-ΔΔCt; 1-way ANOVA).

## Slide 3
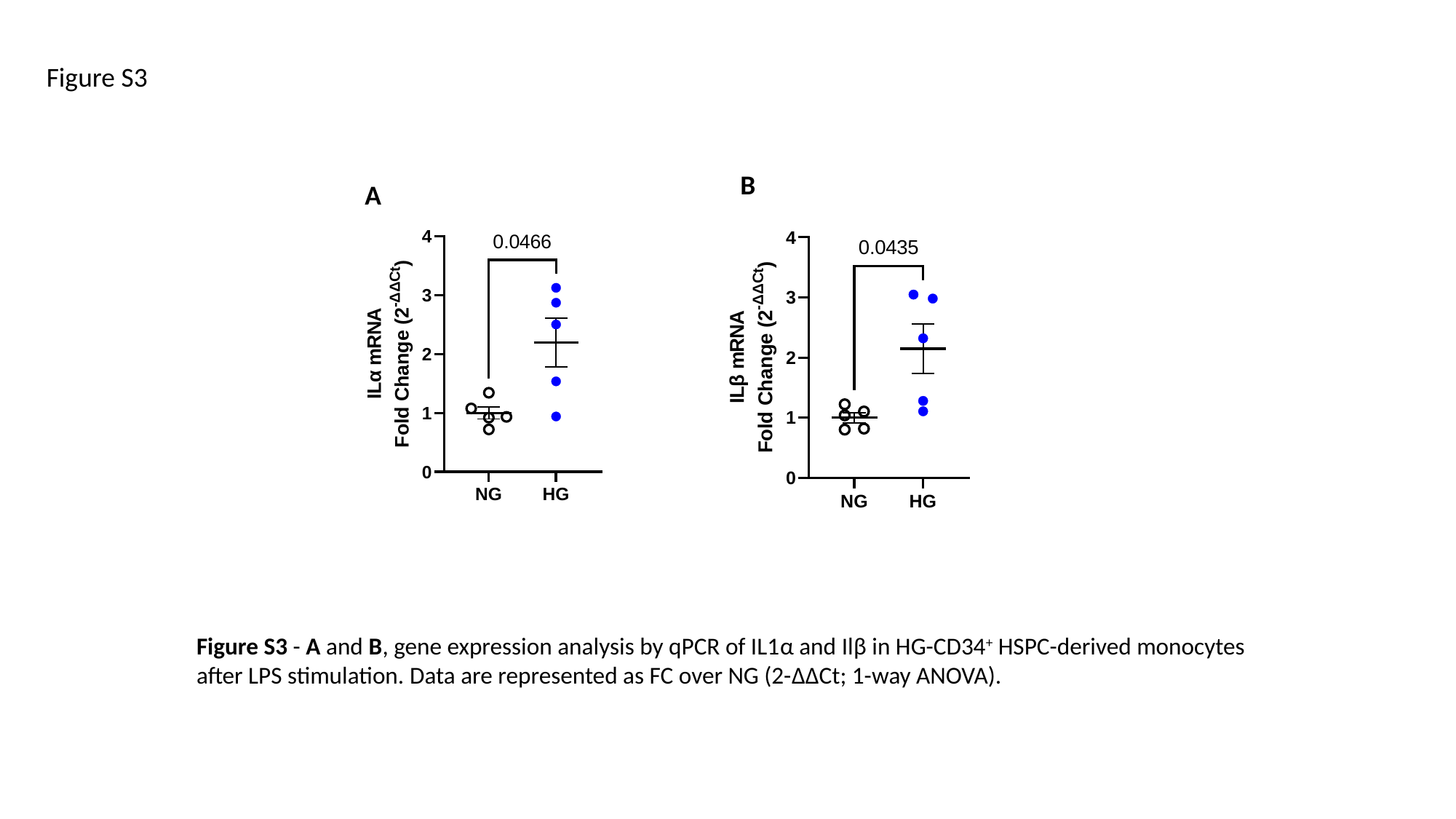

Figure S3
B
A
Figure S3 - A and B, gene expression analysis by qPCR of IL1α and Ilβ in HG-CD34+ HSPC-derived monocytes after LPS stimulation. Data are represented as FC over NG (2-ΔΔCt; 1-way ANOVA).
