# Supplementary material for: Persistent epigenetic signals propel a senescence-associated secretory phenotype and trained innate immunity in CD34+ hematopoietic stem cells from diabetic patients
Source: Cardiovasc Diabetol. 2024 Mar 29;23:107. doi: 10.1186/s12933-024-02195-1 (PMC10981360; doi:10.1186/s12933-024-02195-1)
Supplement: Supplementary file 2 — Additional file 2: Table S1. Primer List for qPCR. Table S2. Antibody list. [file 12933_2024_2195_MOESM2_ESM.docx]

Table S1: Primer List for qPCR

| **Name** | **Forward Primer** | **Reverse Primer** |
| --- | --- | --- |
|  | **RT-PCR** |  |
| p21 | GGAAGACCATGTGGACCTGT | GGATTAGGGCTTCCTCTTGG |
| p27 | ACCTGCAACCGACGATTCTTC | GGGCGTCTGCTCCACAGA |
| IL6 | ACAAAAGTCCTGATCCAGTTCC | GACTGCAGGAACTCCTTAAAGC |
| TNFα | CCCAGGGACCTCTCTCTAATCA | AGCTGCCCCTCAGCTTGAG |
| NFkB-p65 | ACCCCTTCCAAGAAGAGCAGCGT | TCTCATCCCCACCGAGGCAGC |
| SetD7 | AACGGTCCAGCCCAGGAATA | AAGGCTTCCTCCATCTGGGT |
| β2-microglobulin | GGACTGGTCTTTCTATCTCTTGTAC | ACCTCCATGATGCTGCTTAC |
| KAT3B | GGGCCGAAGAAGAGATTTCCTG | GAGCCAAAATCTGTGCCATCG |
| IL1α | GCGTTTGAGTCAGCAAAGAAGTC | ATGCAGCCTTCATGGAGTGG |
| IL1β | CAGCTACGAATCTCCGACCAC | GGCAGGGAACCAGCATCTTC |
|  | **ChIP** |  |
| NFkB-p65 | GGTGGTGGCCCCTTGAGTTT | GGCCGACTTCAGGTGACAGA |
|  | **Telomere** |  |
| Human 36B4F | CAGCAAGTGGGAAGGTGTAATCC | CCCATTCTATCATCAACGGGTACAA |
| Human b-globin | GCTTCTGACACAACTGTGTTCACTAGC | CACCAACTTCATCCACGTTCACC |

Table S2: Antibody List

| **Antibody** | **Provider** | **Clone or Antiantibody ID** | **WB Dilution** | **ChiP** | **MWt. (kDa)** |
| --- | --- | --- | --- | --- | --- |
| NFkB-p65 | Cell signaling | 8242 | 1:1000 |  | 65 |
| Acetyl K310 NFkB-p65 | Cell signaling | 3045 | 1:1000 |  | 65 |
| Monoclonal anti-β-actin  HRP-conjugate | Sigma-Aldrich | A3854 | 1:10000 |  | 42 |
| Goat anti-Rabbit IgG-HRP | Thermo Fisher Scientific | G-21234 | 1:5000 |  |  |
| Tri-Methyl-Histone H3 (Lys9) antibody - ChIP, Rabbit | Cell signaling | 13969 |  | 5ug |  |
| Mono-Methyl-Histone H3 (Lys4) antibody - ChIP, Rabbit | Cell signaling | 5326 |  | 5ug |  |
| SETD7 antibody, Rabbit | Cell Signaling | 2813 |  | 5ug |  |
| RNA POL II (Rpb1 NTD) | Cell Signaling | 14958S |  | 5ug |  |
| anti-rabbit AlexaFluor488 | Thermo Fisher Scientific | A-11008 | 1:1000 |  |  |
| p21 Waf1/Cip1 (12D1) Rabbit mAb | Cell signaling | 2947 | 1:1000 |  |  |
| p27 Kip1 (D69C12) XP® Rabbit mAb | Cell signaling | 3686 | 1:1000 |  |  |
