# Supplementary material for: Persistent epigenetic signals propel a senescence-associated secretory phenotype and trained innate immunity in CD34+ hematopoietic stem cells from diabetic patients
Source: Cardiovasc Diabetol. 2024 Mar 29;23:107. doi: 10.1186/s12933-024-02195-1 (PMC10981360; doi:10.1186/s12933-024-02195-1)
Supplement: Supplementary file 3 — Additional file 3: Additional material & methods. [file 12933_2024_2195_MOESM3_ESM.docx]

**CD34^+^ HSPC growth curves**

CD34^+^ stem cells were seeded at an initial density of 2x${10}^{5}$ cells/wells, and cultured for up to 20 days in HG and NG conditions. Cells were counted on days 5, 10, 15 and 20.

**Myeloid Differentiation**

The CD34^+^ stem cells were differentiated following the protocol of Stec et al.^15^. Briefly, after 20 days amplification in NG and HG condition stem cells were harvested and seeded at a concentration of 1x10^6^ cells/wells in IMDM medium (Iscove's Modified Dulbecco's Medium, Lonza), with 10% FBS and the cytokines SCF (25 ng/mL), IL3 (30 ng/mL), FLT-3 (30 ng/mL) e M-CSF (Macrophage colony-stimulating factor, 30 ng/mL). After 14 days, the cells were harvested for analysis.

**Flow Cytometry**

CD34^+^ stem cells were stained with FITC Annexin V Apoptosis Detection Kit I (BD Biosciences), and with CellROX™ Green Reagent, (Invitrogen™-Thermo Fisher Scientific) for apoptosis and oxidative stress detection respectively. Antihuman APC CD14 (BD Biosciences) and antihuman PE CD16 (BD Biosciences) were used for the characterization of monocyte subpopulations. The Gallios Flow Cytometer (Beckman Coulter) and Kaluza analysis software were used to analyze samples using appropriate physical gating. At least ${10}^{4}$ events in the indicated gates were acquired.

**RNA extraction, cDNA Preparation and Quantitative Polymerase Chain Reaction**

Total RNA from CD34^+^ stem cells were isolated using Direct-Zol RNA kit (Zymo Research, Irvine, CA), following the manufacturer’s protocols. The RNA concentration and quality were assessed by microvolume spectrophotometry using ND-1000 Nanodrop (Thermo Fisher Scientific) and by electrophoresis. According to the manufacturer's protocol, total RNA (500 ng) was reverse transcribed into cDNA with the Superscript III kit (Invitrogen™- Thermo Fisher Scientific) cDNA was used to quantify gene expression. The data obtained were normalized to each housekeeping gene and then expressed as fold-change ($2^{-\Delta\Delta Ct}$) over NG. Primers are reported in Table S1. All reactions were performed with SYBR Green Supermix 2X (Bio-Rad Laboratories) on CFX96 Real–Time System PCR (Bio-Rad).

**DNA extraction and telomere length assessment**

Genomic DNA was isolated by the PureLink Genomic DNA kit (Invitrogen™-Thermo Fisher Scientific), following the manufacturer's protocols. Nucleic acid samples were quantified by NanoDrop, and integrity was analyzed by 1% agarose gel electrophoresis. Telomere length was calculated by qPCR with primers for telomeric regions and β-globin as internal reference (Table S1). The relative telomere length expressed as fold-change ($2^{-\Delta\Delta Ct}$) over NG^16^.

**Chromatin Immunoprecipitation Assays**

Treated cells were washed and cross-linked using 2% formaldehyde for 8 minutes at 37°C. After stopping cross-linking by adding 2.5 M of glycine, cell lysates were sonicated with Episonic 2000 (Epigentek Group Inc.) and centrifuged at 4°C for 10 minutes at 14000 rpm. Supernatants were immunoprecipitated using the EpiTect ChIP OneDay kit (Qiagen), following the manufacturer’s protocols. The list of antibodies used is reported in Table S2. Recovered DNA fragments were amplified for the NFKB-p65 promoter gene by qPCR (Table S1). qPCR values were normalized to input DNA and to the values obtained with immunoglobulin G isotype. The data are expressed as NG percentages.

**Western Blot analysis**

Cells were lysed on ice in RIPA buffer and after BCA protein quantification (Pierce™-Thermo Fisher Scientific), 50 µg of proteins in Laemli buffer 6X were run on acrylamide 10% gel. Proteins were transferred on PVDF membrane (Millipore, Merck Spa) and incubated with the relevant primary antibody overnight at 4°C in agitation. After washing, membranes were incubated with the secondary antibody linked to horseradish peroxidase (Invitrogen™-Thermo Fisher Scientific) and revealed by ECL Western Blotting Substrate (Pierce™-Thermo Fisher Scientific). Densitometric analysis was performed with the software Alliance 9.7 Western Blot Imaging System 8 (Uvitec Ltd). Proteins’ levels were normalized according to the β-actin signal. All antibodies used are reported in the Table S2.

**ELISA Assay**

Conditioned media from UCB-derived CD34^+^ HSPCs and monocytes were collected after 72 and 20 hours respectively. Supernatants were analyzed for pro-inflammatory cytokines IL-6 and TNF-α by ELISA Assay using the specific Kit (Invitrogen™-Thermo Fisher Scientific), according to the manufacturer’s instruction. Cytokine concentration extrapolated from the standard curve was normalized on the total number of plated cells.

**Nuclear NFkB-p65 quantification**

CD34^+^HSPCs were harvested and treated with the Fixation/Permeabilization solution kit (BD Cytofix/Cytoperm) for 30 min in agitation at 4°C. After two washes in Perm/Wash Buffer, cells were resuspended in staining buffer (PBS, 5 mM EDTA, 0.1% BSA) and incubated for 20 minutes at RT in agitation with anti-human NFκB-p65 (Cell Signaling). After washing, secondary antibody anti-rabbit AlexaFluor488 (Invitrogen™-Thermo Fisher Scientific) was added for 15 minutes at RT. Nuclei were counterstained with DRAQ5 and the fluorescence of the samples was measured with the Amnis ImageStream®X Mk II device (Amnis Corporation, Austin, TX, USA) with 40 magnification and low flow rate/high sensitivity using the INSPIRE ImageStreamX MkII software. Data from 10,000 events per sample were collected, and the fluorescence overlay between NFkB-p65 and DRAQ5 was calculated using the IDEA 6.2 software and expressed as Similarly Dilate Index (Amnis Corporation).

**NFkB-p65 Binding Activity**

The DNA binding reaction was carried as previously reported ^17^. Briefly, 10ug of the protein from each condition was incubated for 1h at RT in a 96-well plate coated with consensus sequences for NFκB (GGGACTTTCC). After washing, the NFκB p65-antibody (Active Motif) was added and incubated for 1 h, followed by incubation with a horseradish peroxidase-conjugated secondary antibody.
